# ^18^F-FDG PET/CT in patients with polymyositis/dermatomyositis: correlation with serum muscle enzymes

**DOI:** 10.1186/s41824-020-00084-w

**Published:** 2020-08-12

**Authors:** Hanae Arai-Okuda, Takashi Norikane, Yuka Yamamoto, Katsuya Mitamura, Kengo Fujimoto, Yasukage Takami, Risa Wakiya, Shusaku Nakashima, Hiroaki Dobashi, Yoshihiro Nishiyama

**Affiliations:** 1 Department of Radiology, Faculty of Medicine, Kagawa University, 1750-1 Ikenobe, Miki-cho, Kita-gun, Kagawa, 761-0793 Japan; 2 Division of Hematology, Rheumatology and Respiratory Medicine, Department of Internal Medicine, Faculty of Medicine, Kagawa University, Kagawa, Japan

**Keywords:** ^18^F-FDG, PET, Polymyositis, Dermatomyositis

## Abstract

**Background:**

Muscle enzymes are the major noninvasive diagnostic parameters useful in polymyositis/dermatomyositis (PM/DM). Few studies have yet correlated findings on ^18^F-FDG PET with disease activity in patients with PM/DM.

**Purpose:**

We evaluated ^18^F-FDG muscle uptake in patients with PM/DM compared with non-muscular diseases and correlated the results with serum muscle enzymes.

**Methods:**

A total of 28 patients with untreated PM/DM and 28 control patients with non-muscular diseases were examined with ^18^F-FDG PET/CT. ^18^F-FDG uptake was evaluated in 9 proximal skeletal muscle regions bilaterally. The uptake was scored as follows: 0 = less than that of the mediastinal blood vessels, 1 = greater than or equal to that of the mediastinal blood vessels, and 2 = greater than or equal to that of the liver. A score 1 or 2 was considered positive. The mean and maximum standardized uptake values (SUV) were calculated in each muscle and were averaged for all muscle regions. PET findings were correlated with serum muscle enzymes.

**Results:**

^18^F-FDG uptake was observed in 82% of patients with PM/DM and 7% of control patients. The number of positive regions, total score, mean SUVmean, and mean SUVmax in patients with PM/DM were significantly higher than those in the control patients (all *P* < 0.001). The total score of 2 was the best cut-off value that could discriminate patients with PM/DM from control patients. The total score, mean SUVmean, and mean SUVmax showed significant correlations with creatine kinase (*P* = 0.047, 0.002, 0.010, respectively) and aldolase (*P* = 0.036, 0.005, 0.038, respectively).

**Conclusion:**

^18^F-FDG PET/CT using visual and SUV methods demonstrated its usefulness by discriminating PM/DM from non-muscular diseases and correlating with serum muscle enzymes in patients with PM/DM.

## Introduction

Polymyositis (PM) and dermatomyositis (DM) are chronic inflammatory diseases that affect systemic skeletal muscles, lungs, and other extramuscular organs (Dalakas and Hohlfeld, 2003). Muscle enzymes such as creatine kinase (CK) and aldolase provide information about myogenic pathologies and are the major noninvasive diagnostic parameters useful in PM/DM. Although invasive, the gold standard in the diagnosis of PM/DM is muscle biopsy. However, because the distribution of muscle lesions in patients with PM/DM is frequently patchy, the sites of inflammation or the global activity and/or severity of myositis may not always be apparent in a biopsy specimen (Dalakas, 1991).

2-deoxy-2-^18^F-fluoro-D-glucose (^18^F-FDG) accumulates not only in malignant lesions but also in inflammatory ones infiltrated by glucose-consuming inflammatory cells (Gotthardt et al., 2010; Vaidyanathan et al., 2015). ^18^F-FDG uptake in proximal muscles in patients with PM/DM exceeds that in controls (Tanaka et al., 2013; Pipitone et al., 2012; Tateyama et al., 2015; Owada et al., 2012). Tanaka *et al*. showed that mean proximal muscle standardized uptake value (SUV) in patients with PM/DM correlated with serum CK and aldolase (Tanaka et al., 2013), whereas some other investigators found no correlation between ^18^F-FDG uptake in proximal muscles and serum CK levels (Pipitone et al., 2012; Tateyama et al., 2015). Few studies have yet correlated findings on ^18^F-FDG positron emission tomography (PET) with disease activity in patients with PM/DM. This prompted us to evaluate ^18^F-FDG uptake in proximal skeletal muscles using visual and SUV methods in PM/DM compared with non-muscular diseases and correlate the results with serum muscle enzymes.

## Materials and methods

### Patients

We conducted a retrospective analysis of part of prospective ^18^F-FDG PET/CT study for diseases not covered by health insurance. From June 2010 to February 2020, 28 patients (6 males, 22 females; mean age, 66 years; age range, 42–77 years) with newly diagnosed PM/DM who underwent ^18^F-FDG PET/CT before receiving initial corticosteroid treatment were included in the study. The diagnosis of PM/DM was based on the criteria of Bohan and Peter (1975), and the patient cohort was limited to those with definite or probable PM/DM. They were identified through a retrospective review of medical records in our hospital. Serum levels of muscle enzymes including CK and aldolase were analyzed by routine laboratory techniques. The same number of age- and sex-matched control patients with non-muscular diseases who had undergone ^18^F-FDG PET/CT was also identified. This study was approved by our institutional ethical review committee. Written informed consent was obtained from PM/DM patients. The requirement for informed consent for control patients was waived.

### PET/CT imaging

^18^F-FDG was produced using an automated synthesis system with HM-18 cyclotron (QUPID; Sumitomo Heavy Industries Ltd, Tokyo, Japan).

All acquisitions were performed using a Biograph mCT 64-slice PET/CT scanner (Siemens Medical Solutions USA Inc., Knoxville, TN, USA). This scanner has an axial field of view of 21.6 cm. Patients were instructed to fast for at least 5 h before ^18^F-FDG administration. A normal glucose level in the peripheral blood was confirmed before the injection. PET emission scanning (2 min per bed position) was performed 90 min after intravenous injection of ^18^F-FDG (5 MBq/kg) from the midcranium to the knee and co-registered with an unenhanced CT of the same region (quality reference mAs, 100 mAs [using CARE Dose4D]; reconstructed slice thickness, 5 mm). The PET data were acquired in three-dimensional mode and reconstructed with a baseline ordered-subset expectation maximization algorithm, incorporating correction with point-spread function and time-of-flight model (2 iterations, 21 subsets). A Gaussian filter with a full-width at half-maximum of 5 mm was used as a post-smoothing filter.

### PET/CT data analysis

At first, images were visually assessed by two board-certified nuclear medicine physicians independently in a blinded manner. Any difference of opinion was resolved by consensus. ^18^F-FDG uptake in proximal skeletal muscles was evaluated in 18 regions (upper arms; shoulders; sternocleido-mastoid muscles; paraspinal muscles of cervical, upper thoracic, lower thoracic and lumbar levels; buttocks and upper part of the thighs, on both sides) mostly based on a previous report (Tateyama et al., 2015) and was scored as follows: 0 = less than that of the mediastinal blood vessels, 1 = greater than or equal to that of the mediastinal blood vessels, and 2 = greater than or equal to that of liver. A score of 1 or 2 was considered positive. The number of positive regions was counted in each patient (number ranged from 0 to 18). The score of 18 muscle regions was also added in each patient (total score ranged from 0 to 36).

Next, a circular region of interest (ROI) (20 mm in diameter) was placed in the highest ^18^F-FDG uptake area in each of the 18 muscle region, excluding regions obviously influenced by ^18^F-FDG uptake in other anatomical structures by a board-certified nuclear medicine physician. The SUV was calculated using the following formula: SUV = *c*_dc_/(*d*_i_/*w*), where *c*_dc_ is the decay-corrected tracer tissue concentration (Bq/g); *d*_i_, the injected dose (Bq); and *w*, the patient’s body weight (g). The mean SUV (SUVmean) and maximum SUV (SUVmax) were calculated in ROI. For patient-based assessment, the mean SUVmean and mean SUVmax were calculated by averaging the values obtained for the 18 muscle regions.

### Statistical analysis

All statistical analyses were performed using a software package (SPSS Statistics, version 26; IBM). Data were analyzed for statistical significance using the Mann-Whitney *U* test and Spearman’s correlation coefficient. Receiver operating curve (ROC) analyses [providing area-under-the-curve (AUC) values] were performed to evaluate the diagnostic ability of the ^18^F-FDG PET/CT parameters to discriminate between PM/DM and control patients. Differences were considered statistically significant at *P* values less than 0.05.

## Results

^18^F-FDG PET/CT findings together with the clinical data for all 28 patients with PM/DM are presented in Table 1. Ten patients were PM and 18 were DM. Twenty-eight age- and sex-matched control patients with non-muscular diseases were also identified. Diagnosis of control patients were malignant tumors (*n* = 26) and inflammatory disease (*n* = 2).

**Table 1:** Clinical data and ^18^F-FDG PET/CT findings for 28 patients with PM/DM

Patient No.	Age (years)/sex	PM/DM	CK (U/l)	Aldolase (U/l)	Number of positive regions	Positive uptake in muscles*									Total score	Mean SUVmean**	Mean SUVmax***
						U	S	M	C	UT	LT	L	B	T			
1	52/F	DM	27	8.5	1									□	2	0.80	1.26
2	68/F	DM	392	5.2	12		□	□	□		□	□	□		16	1.29	1.88
3	55/F	DM	83	10.6	1		□								2	0.85	1.33
4	58/F	DM	443	7.8	6	□	□		□						6	1.17	1.69
5	59/F	DM	45	4.6	0										0	0.79	0.94
6	77/F	DM	174	20.1	0										0	0.79	0.91
7	71/M	DM	270	27.6	18	□	□	□	□	□	□	□	□	□	29	1.92	2.70
8	44/F	DM	79	8.1	15	□	□	□		□	□	□	□	□	29	1.48	2.17
9	68/F	PM	1692	15.8	13	□	□	□	□		□		□	□	13	1.39	1.78
10	56/F	DM	3284	31.6	17	□	□	□	□	□	□	□	□	□	23	1.41	1.82
11	46/M	DM	2583	18.1	12	□	□	□	□				□	□	20	1.87	2.83
12	53/F	DM	3329	38.0	12	□	□	□			□		□	□	20	1.93	2.68
13	69/F	PM	3946	59.5	14	□	□	□	□	□			□	□	22	1.58	1.92
14	67/F	PM	362	9.8	0										0	1.01	1.24
15	68/F	PM	2811	33.2	4								□	□	4	1.19	1.44
16	70/F	PM	1481	18.8	6		□						□	□	6	1.03	1.31
17	58/M	PM	75	6.3	0										0	0.75	0.90
18	65/F	DM	1302	25.2	2								□	□	2	0.95	1.15
19	47/F	DM	660	19.4	18	□	□	□	□	□	□	□	□	□	35	1.46	2.03
20	64/M	PM	2029	42.6	18	□	□	□	□	□	□	□	□	□	29	1.83	2.29
21	70/F	PM	236	5.8	18	□	□	□	□	□	□	□	□	□	21	1.51	2.16
22	70/F	PM	215	9.1	16	□	□	□	□	□	□		□	□	26	1.40	1.84
23	43/F	DM	37	8.2	0										0	0.74	0.90
24	42/M	DM	92	8.1	15	□	□	□	□	□	□		□	□	21	1.33	1.80
25	63/F	DM	3207	53.1	18	□	□	□	□	□	□	□	□	□	36	2.00	2.70
26	59/M	DM	354	11.1	18	□	□	□	□	□	□	□	□	□	32	1.68	2.35
27	58/F	PM	601	24.0	18	□	□	□	□	□	□	□	□	□	34	2.03	2.90
28	60/F	DM	210	18.4	18	□	□	□	□	□	□	□	□	□	24	1.51	1.91

*The regions of positive ^18^F-FDG at least in either side of the region are indicated by open squares

**Average SUVmean obtained for the 18 muscle regions

***Average SUVmax obtained for the 18 muscle regions

*PM/DM* polymyositis/dermatomyositis, *CK* creatine kinase, *SUVmean* mean standardized uptake value, *SUVmax* maximum standardized uptake value, *U* upper arms, *S* shoulders, *M* sternocleido-mastoid muscles, *C* paraspinal muscles of cervical level, *UT* paraspinal muscles of upper thoracic level, *LT* paraspinal muscles of lower thoracic level, *L* paraspinal muscles of lumbar level, *B* buttocks, *T* upper part of the thighs

### ^18^F-FDG uptake in patients with PM/DM and control patients

**Fig. 1:**
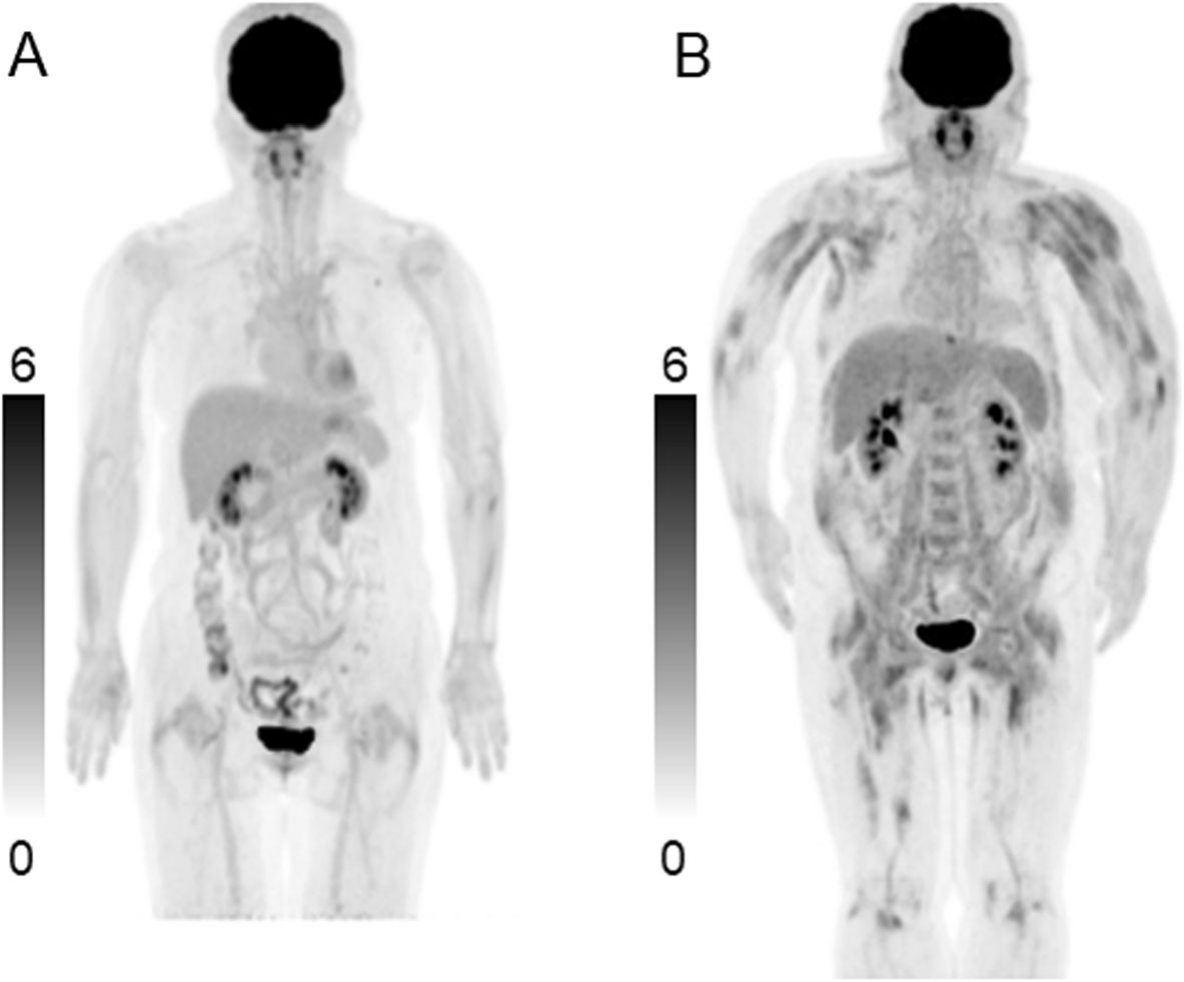
^18^F-FDG PET maximum intensity projection (MIP) image of a 58-year-old female as a control patient shows no increased uptake in the skeletal muscles (**a**). ^18^F-FDG PET MIP image of a 53-year-old female with dermatomyositis shows diffuse uptake in the proximal skeletal muscles (**b**).

Increased ^18^F-FDG uptake in proximal skeletal muscles in least one region was observed in 23/28 (82%) in patients with PM/DM. ^18^F-FDG muscle uptake showed an almost symmetrical distribution. The shoulders, buttocks, and upper part of the thighs were the most frequent ^18^F-FDG-positive regions. In contrast, only 2 of 28 control patients (7%) showed increased ^18^F-FDG uptake in proximal skeletal muscles. Representative ^18^F-FDG PET images in a control patient and a patient with DM are shown in Fig. 1.

Table 2 shows the results of ^18^F-FDG PET/CT parameters in patients with PM/DM and in control patients. The numbers of positive regions, total score, mean SUVmean, and mean SUVmax in patients with PM/DM were significantly higher than those in control patients (all *P* < 0.001).

**Table 2:** ^18^F-FDG PET/CT findings in patients with PM/DM and in control patients

PET/CT parameter	PM/DM (*n* = 28)	Controls (*n* = 28)	*P* value
Number of positive regions	10.4 ± 7.4	0.1 ± 0.4	< 0.001
Total score	16.1 ± 12.8	0.1 ± 0.4	< 0.001
Mean SUVmean	1.35 ± 0.42	0.96 ± 0.76	< 0.001
Mean SUVmax	1.81 ± 0.62	0.97 ± 0.09	< 0.001

Values are mean ± SD

*PM/DM* polymyositis/dermatomyositis, *SUVmean* mean standardized uptake value, *SUVmax* maximum standardized uptake value

There was a significant correlation between the number of positive regions and mean SUVmean (*ρ = 0.791, P* < 0.001) and mean SUVmax (*ρ = 0.872, P* < 0.001). There was also a significant correlation between total score and mean SUVmean (*ρ = 0.791, P* < 0.001) and mean SUVmax (*ρ = 0.879, P* < 0.001).

**Fig. 2:**
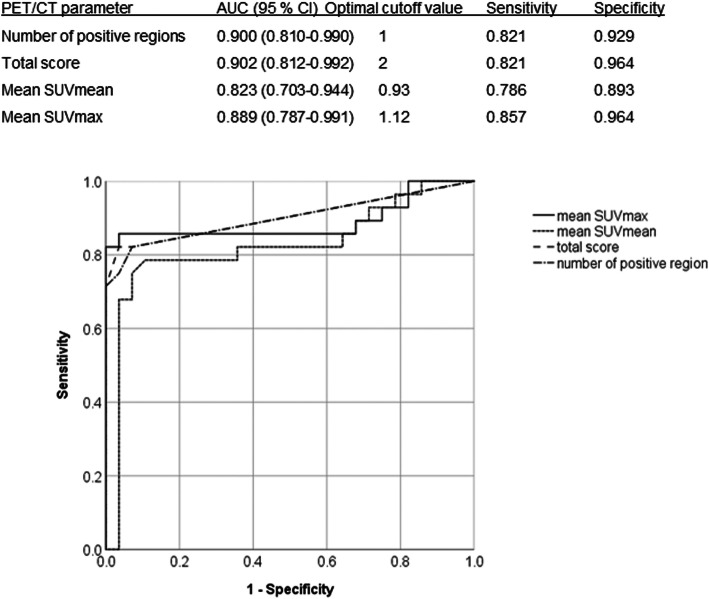
Receiver operating curve analysis for discriminating patients with PM/DM from control patients using ^18^F-FDG PET/CT parameters.

ROC analysis and AUCs of ^18^F-FDG PET/CT parameters for discrimination of patients with PM/DM from control patients are shown in Fig. 2. The total score of 2 was the best cut-off value in discriminating patients with PM/DM from control patients.

### ^18^F-FDG uptake and serum muscle enzymes in patients with PM/DM

Table 3 shows the results of correlation of ^18^F-FDG PET/CT parameters with serum muscle enzymes in patients with PM/DM. The total score, mean SUVmean, and mean SUVmax all showed significant correlations with CK (*P* = 0.047, 0.002, 0.010, respectively) and aldolase (*P* = 0.036, 0.005, 0.038, respectively). The number of positive regions did not show a significant correlation with CK or aldolase.

**Table 3:** Spearman’s correlation coefficient of ^18^F-FDG PET/CT findings with serum muscle enzymes in patients with PM/DM

	Serum muscle enzyme			
	Creatine kinase		Aldolase	
PET/CT parameter	*ρ*	*P* value	*ρ*	*P* value
Number of positive regions	0.354	0.065	0.349	0.069
Total score	0.379	0.047	0.399	0.036
Mean SUVmean	0.575	0.002	0.521	0.005
Mean SUVmax	0.478	0.010	0.395	0.038

*PM/DM* polymyositis/dermatomyositis, *SUVmean* mean standardized uptake value, *SUVmax* maximum standardized uptake value, *ρ* Spearman’s correlation coefficient

## Discussion

The present findings demonstrated that in patients with PM/DM ^18^F-FDG PET/CT visual and SUV parameters were useful in discriminating PM/DM from non-muscular diseases as well as showing correlations with serum muscle enzymes.

Kubota et al. demonstrated that a substantial component of ^18^F-FDG uptake in tumor tissue was due to activity localizing in macrophages, young granulation tissue, and other peri-tumoral inflammatory cell elements with greater ^18^F-FDG uptake than tumor cells (Kubota et al., 1992). We found that 82% patients with PM/DM had ^18^F-FDG-positive muscle regions, whereas 93% of the control patients had ^18^F-FDG-negative muscle regions. Pipitone and colleagues considered ^18^F-FDG muscle uptake not to be specific for myositis, but rather to likely reflect the intensity of metabolic activity within the affected muscles including the contribution of infiltrating inflammatory cells (Pipitone et al., 2012). Tateyama and Owada and their respective coworkers showed increased ^18^F-FDG muscle uptake in 60.6% and 33% of patients with PM/DM (Tateyama et al., 2015; Owada et al., 2012). Compared to Tateyama’s and the present studies, the low sensitivity of ^18^F-FDG PET documented by Owada et al. may have been due to the strict criterion they used for muscle ^18^F-FDG uptake, namely the liver, as a positivity criterion (Owada et al., 2012). In Tateyama’s and the present studies, the mediastinal blood vessels were chosen instead. We found that the number of positive regions, total score, mean SUVmean, and mean SUVmax in patients with PM/DM were significantly higher than those in control patients. These results are consistent with those of two previous reports (Tanaka et al., 2013; Pipitone et al., 2012). Tateyama et al. demonstrated that mean SUVmean and mean SUVmax in patients with PM/DM were significantly higher than those in patients with amyotrophic lateral sclerosis with similar disabilities (Tateyama et al., 2015). This highlights the potential unique and useful role that ^18^F-FDG PET may be able to play in the diagnosis of PM/DM.

PM/DM is clinically characterized by symmetrical proximal muscle weakness (Engel and Hohlfeld, 2004). In the present study, ^18^F-FDG muscle uptake mostly showed a symmetrical distribution in patients with PM/DM, consistent with the results of two previous reports (Tateyama et al., 2015; Owada et al., 2012). These findings statistically verified that the inflammatory muscle damage progresses symmetrically in PM/DM, although muscle lesions are often multifocal in each muscle. Although the exact mechanisms underlying the symmetrical proximal muscle weakness in these patients are unknown, some possible mechanisms that may explain it are involvement of some anatomical factors, including blood vessels and peripheral nerves, or immune or physiological factors of individual muscles that can influence the extent of inflammation (Tateyama et al., 2015).

Although serum CK levels are a major clinical biomarker in PM/DM, they do not always reliably reflect disease activity (Rider and Miller, 1995). Tanaka et al. documented that mean SUVmean correlated with serum CK and aldolase levels (Tanaka et al., 2013). Conflicting results have also been obtained regarding the relation between ^18^F-FDG SUV parameters and serum CK levels (Pipitone et al., 2012; Tateyama et al., 2015). In the present study, total score, mean SUVmean and mean SUVmax were all significantly correlated with serum CK and aldolase levels. This discrepancy in the significance of ^18^F-FDG PET parameters may be attributable to differences in different aspects of myositis and/or differences in the imaging protocols and image analyses used in the individual studies. Tateyama’s study included patients shortly after the beginning of corticosteroid therapy (Tateyama et al., 2015). To date, there is still very limited experience of ^18^F-FDG PET and serum muscle enzyme measurements in patients with PM/DM. Further studies are needed to evaluate the correlation between ^18^F-FDG PET findings and disease biology in these patients.

Limitations of the present study include small sample size and retrospective design. Magnetic resonance imaging (MRI) findings and muscle biopsy data were not obtained from all patients, and so comparisons of their results and ^18^F-FDG PET/CT findings were not possible. The most important advantage of ^18^F-FDG PET/CT is that it can screen the whole body in one scan. We can evaluate the extent of muscle lesions systemically at one time including sites that are not routinely screened by MRI. However, as is well known, ^18^F-FDG uptake in muscles is influenced by hyperglycemia, uptake by other organs, and voluntary or involuntary muscle movement during the uptake phase (Jackson et al., 2006); therefore, the examination must be conducted under strict conditions.

^18^F-FDG PET/CT also offers additional benefits in the evaluation of patients with PM/DM. The increased prevalence of malignant tumors in these patients has been well documented (Bohan and Peter, 1975), which necessitates screening investigations at the time of PM/DM diagnosis. ^18^F-FDG PET/CT can be also useful in determining the activity of interstitial lung disease, which is a possibly critical complication in PM/DM (Owada et al., 2012). In a case report by Renard et al., ^18^F-FDG muscle uptake in a patient with DM after immunosuppressive therapy nearly normalized (Renard et al., 2012). Further additional large prospective studies are needed to confirm our results and their potential clinical value in patients with this difficult to characterize disease.

## Conclusion

These preliminary results suggested that ^18^F-FDG PET/CT using visual and SUV methods showed its usefulness by discriminating PM/DM from non-muscular diseases and correlating with serum muscle enzymes in patients with PM/DM.

## Data Availability

All datasets used during the current study are available from the corresponding author on reasonable request.

## Abbreviations

AUC: Area-under-the-curve
CK: Creatine kinase
DM: Dermatomyositis
^18^F-FDG: 2-deoxy-2-^18^F-fluoro-D-glucose
MRI: Magnetic resonance imaging
PET: Positron emission tomography
PM: Polymyositis
ROC: Receiver operating curve
ROI: Region of interest
SUV: Standardized uptake value
SUVmax: Maximum SUV
SUVmean: Mean SUV
